# Global transcriptional responses of pneumococcus to human blood components and cerebrospinal fluid

**DOI:** 10.3389/fmicb.2022.1060583

**Published:** 2022-12-21

**Authors:** Jens Sivkær Pettersen, Frida Fabricius Høg, Flemming Damgaard Nielsen, Jakob Møller-Jensen, Mikkel Girke Jørgensen

**Affiliations:** Department of Biochemistry and Molecular Biology, University of Southern Denmark, Odense, Denmark

**Keywords:** *Streptococcus pneumoniae* (pneumococcus), RNA-sequencing, gene regulation and expression, small non-coding RNAs, human plasma and cerebrospinal fluid, sepsis

## Abstract

*Streptococcus pneumoniae* (pneumococcus) is a leading cause of severe invasive infectious diseases such as sepsis and meningitis. Understanding how pneumococcus adapts and survive in the human bloodstream environment and cerebrospinal fluid (CSF) is important for development of future treatment strategies. This study investigates the global transcriptional response of pneumococcus to human blood components and CSF acquired from discarded and anonymized patient samples. Extensive transcriptional changes to human blood components were observed during early stages of interaction. Plasma-specific responses were primarily related to metabolic components and include strong downregulation of fatty acid biosynthesis genes, and upregulation of nucleotide biosynthesis genes. No transcriptional responses specific to the active plasma proteins (e.g., complement proteins) were observed during early stages of interaction as demonstrated by a differential expression analysis between plasma and heat-inactivated plasma. The red blood cell (RBC)-specific response was far more complex, and included activation of the competence system, differential expression of several two-component systems, phosphotransferase systems and transition metal transporter genes. Interestingly, most of the changes observed for CSF were also observed for plasma. One of the few CSF-specific responses, not observed for plasma, was a strong downregulation of the iron acquisition system *piuBCDA*. Intriguingly, this transcriptomic analysis also uncovers significant differential expression of more than 20 small non-coding RNAs, most of them in response to RBCs, including small RNAs from uncharacterized type I toxin-antitoxin systems. In summary, this transcriptomic study identifies key pneumococcal metabolic pathways and regulatory genes involved with adaptation to human blood and CSF. Future studies should uncover the potential involvement of these factors with virulence *in-vivo*.

## Introduction

The gram-positive bacterium *Streptococcus pneumoniae* (pneumococcus) is an opportunistic pathogen and a worldwide leading cause of severe infectious diseases such as pneumonia, sepsis, and meningitis. Fortunately, in recent years, the overall rate of invasive pneumococcal diseases (IPD) has declined in many countries due to the introduction of the pneumococcal conjugate vaccines (PCV7; PCV13; Harboe et al., 2014; Waight et al., 2015). The mortality rate, however, remains high (~20%; Chen et al., 2021). In many developing countries where the PCVs are not widely introduced into the immunization programs, the burden of pneumococcal disease is still very high – especially among children younger than 5 years (O’Brien et al., 2009; Troeger et al., 2018). On top of this, with the emergence of non-vaccine serotypes and antibiotic resistant strains, *S. pneumoniae* continues to be a major public health issue worldwide (Slotved et al., 2016; Cillóniz et al., 2018; Ouldali et al., 2021). For these reasons, continuous effort to understand its pathogenicity and ability to survive in different host niches is of great importance for development of future treatment strategies.

The ability of pneumococcus to invade and survive in the most lethal invasive niches, such as the bloodstream and meninges, has been broadly investigated in the past (Hava and Camilli, 2002; Orihuela et al., 2004; Hyams et al., 2010a; Iovino et al., 2016a). However, when it comes to the specific transcriptional responses and the regulatory mechanisms that control the adaptation to these environments, there is still a lot to learn.

The route of pneumococcus to an invasive infection starts from its primary colonization site in the nasopharynx. From here, the pneumococci may spread locally to the middle ear, causing otitis media, or to the lung by aspiration, causing pneumonia (Weiser et al., 2018). Invasive infection of the bloodstream and meninges further requires that pneumococcus breaches the epithelial and endothelial barriers, which is possible through multiple routes and mechanisms (Ring et al., 1998; Zhang et al., 2000; Iovino et al., 2016b). Irrespective of the route, once inside the bloodstream, the pneumococcus must overcome different challenges to adapt to the new host environment and sustain an infection. These challenges include nutrient availability and the host immune system (Paterson and Orihuela, 2010; Honsa et al., 2013). Splenic macrophages, neutrophils, and complement-mediated opsonophagocytosis are in particularly important aspects of the immune system involved with pneumococcal clearance in the blood (Brown et al., 1981; Paterson and Orihuela, 2010). Nevertheless, pneumococcus is a master of disguise and expresses an immunoprotective polysaccharide capsule and complement-interfering proteins such as Pneumococcal surface protein A and C (PspA and PspC) and Pneumolysin (Ply), which enables pneumococcus to escape recognition by the host immune system (Tu et al., 1999; Yuste et al., 2005; Hyams et al., 2010b).

The bloodstream is one of the major routes of pneumococcus to reach the meninges. Studies indicate that pneumococcus invades the meninges through receptor-mediated transcytosis across the blood–brain barrier, either *via* PAFR, pIgR, or PECAM-1 (Iovino et al., 2016b). Once inside subarachnoid space of the meninges, pneumococcus starts replicating, which eventually leads to the recruitment of leukocytes, and the onset of meningitis (Mook-Kanamori et al., 2011). To replicate and survive in meninges, it is evident that pneumococcus needs to harness nutrients available in the cerebrospinal fluid (CSF) or from surrounding cells. CSF is secreted by the choroid plexus and its compositions closely resembles an ultrafiltrate of plasma, with some of the main differences being that CSF contains substantially lower levels of proteins and fatty acids, and slightly lower levels of glucose (Jumpertz et al., 2012; Hladky and Barrand, 2014).

It is evident that reprogramming of virulence gene expression is crucial for survival and adaptation to the above-mentioned host niches (Polissi et al., 1998; Hava and Camilli, 2002). Therefore, we decided to investigate how the pneumococci transcriptionally adapt to interaction with human blood components and CSF, which are two of the major components that pneumococci face during invasive infections. A few studies have previously used mice models, or niche-mimicking conditions, to study the transcriptional responses during invasive infections (Orihuela et al., 2004; Ogunniyi et al., 2012; Shenoy et al., 2017; Aprianto et al., 2018; D’Mello et al., 2020). However, to gain further insight into human-specific interactions, this study focuses on human-derived samples. While *in-vivo* infection models certainly have their advantages when it comes to imitating the complexity of the host, using a more simplistic *in-vitro* model allow us to decipher the transcriptional responses caused by the specific components present in these human body fluids.

Using RNA-sequencing, this study uncovers extensive and immediate transcriptional changes of the pathogenic *S. pneuomniae* D39V strain during contact with human plasma, red blood cells (RBCs) and CSF. We show that the pneumococcal transcriptional response to heat-inactivated plasma is no different from normal plasma, suggesting that the active host plasma proteins, such as the complement proteins, are not sensed by pneumococcus – at least not in in the early stages of a bloodstream infection. Additionally, the transcriptional response to plasma is largely a metabolic response, involving strong regulation of fatty acid biosynthesis and *de novo* nucleotide biosynthesis, whereas the response to the RBCs is more complex, and involves transcriptional regulation of approximately a quarter of all pneumococcal genes.

To further understand regulatory networks involved with adaptation to human blood and CSF, we also investigated the potential involvement of regulatory small non-coding RNAs (sRNAs), which are scarcely investigated in the field of pneumococcal gene regulation. More than 100 sRNAs have so far been identified, but only a very few have been characterized (Mann et al., 2012; Sinha et al., 2019). Interestingly, 22 of these sRNAs are significantly differentially expressed across the different conditions from this study. Many of these are uncharacterized sRNAs and require further investigation to uncover their function and involvement with gene regulation during adaptation to human blood and CSF.

## Materials and methods

### Strains and growth conditions

The strains used in this study were the serotype 2 strain *Streptococcus pneumoniae* D39V (Slager et al., 2018a) and the serum-sensitive *Escherichia coli* NU14. For *S. pneumoniae*, all liquid growth was carried out in a chemically defined medium (CDM), as defined by Kloosterman et al. (2006), at 37°C with 5% CO_2_. Depending on the experiment, CDM was supplemented with 20% human plasma, red blood cell suspension or cerebrospinal fluid. For colony-forming unit (CFU) assays, serial dilutions of *S. pneumoniae* was plated on Columbia agar plates supplemented with 2% (v/v) defibrillated horse-blood and incubated over-night at 37°C with 5% CO_2_. For plasma-survival assays, over-night cultures of *E. coli* NU14 grown in in Luria-Bertani (LB) broth at 37°C were used to inoculate cultures of 50% LB broth and 50% plasma or heat-inactivated plasma. For *E. coli* NU14 CFU measurements, serial dilutions were plated on Columbia agar plates and incubated over-night at 37°C.

### Collection of human samples

Human blood samples, fractionated into plasma and red blood cell fractions were acquired from healthy blood donors from Odense University Hospital (OUH) in Odense, Denmark (project No. DP077). Cerebrospinal fluid (CSF) samples were obtained as leftover material from routine analysis of patient samples. The samples were obtained fully anonymized, transferred to unlabeled tubes and pooled. In total, samples from 28 donors were pooled for both blood and CSF samples. The pooled samples were then used for growth of bacteria. The use of leftover patient material for this purpose has been evaluated by the Regional Committees on Health Research Ethics for Southern Denmark (S-20212000 Acadre 21/35007). One part of the pooled plasma sample was heat-inactivated at 56°C for 30 min for testing the effect of plasma proteins (e.g., complement proteins) on growth and transcriptional response of pneumococcus.

### RNA extraction

Extraction of RNA was carried out largely as described previously (Andreassen et al., 2018). Briefly, total RNA for RNA-sequencing was extracted from cultures terminated in liquid nitrogen. Cell pellets were resuspended in 150 μl cold solution 1 (10 mM Na-Citrate, 10 mM Na-acetate pH 4.5. and 2 mM EDTA) and added to a mixture of 150 μl solution 2 (10 mM Na-acetate pH 4.5, and 2% SDS), 700 μl acidic phenol (pH 4.5) and 300 μl chloroform. Tubes were inverted and heated to 80°C followed by brief cooling on ice, and spun at 10,000 × *g* for 5 min. The aqueous phase was removed and precipitated in 96% ethanol with Na-acetate. RNA was pelleted, washed once in 70% ethanol, twice in 96% ethanol and resuspended in H_2_O.

Specifically for the cultures incubated with RBCs, RNA extraction included an additional, initial centrifugation step at 250 x g for 5 min at 4°C to separate the RBCs from *S. pneumoniae*.

### RNA-sequencing and data analysis

D39V WT was inoculated in triplicates to a start OD_600_ of 0.15–0.2 in CDM, CDM with 20% plasma, CDM with 20% heat-inactivated plasma, CDM with 20% RBC suspension, and CDM with 20% CSF, and grown for 15 and 45 min before RNA extraction. Ribosomal RNA depletion was carried out using NEBNext rRNA Depletion Kit (Bacteria; New England BioLabs), and subsequent RNA-seq library preparation was carried out with NEBNext Ultra II Directional RNA Library Prep Kit for Illumina (New England Biolabs). Library quality was assessed using the Fragment Analyzer. Paired-end sequencing was carried out on a NovaSeq 6,000 System (Illumina).

The raw paired-end reads were mapped to the *S. pneumoniae* D39V chromosome (GenBank: CP027540.1) with Bowtie2 using local alignment and quantified using featureCounts from the Subread package. Recently identified small RNAs from D39W (Sinha et al., 2019) were manually added to the D39V Genome Feature Format (gff) file to allow for a more elaborate analysis of differentially expressed non-coding RNAs in *S. pneumoniae* D39. Reads were assigned to features with largest overlap, and multimapping reads were counted using a fractional count (1/n, where n is the total number of alignments reported). Differential expression analysis was performed in R using EdgeR. Kyoto Encyclopedia of Genes and Genomes (KEGG) pathway enrichment analysis was performed *via* the Bioconductor package *clusterProfiler* (Yu et al., 2012; Wu et al., 2021), while heatmaps were made with the *pheatmap* package.

### Northern blotting

Northern blot analysis was carried out as previously described (Andreassen et al., 2018). Briefly, 10 μg of RNA was run on a denaturing 8% polyacrylamide gel for 2 h at 300 V. Separated RNA was blotted onto a Zeta-probe nylon membrane (Bio Rad) using a semi-dry transfer unit for 1 h at 400 mA. RNA was cross-linked to the membrane with UV radiation. Oligonucleotide probes (see Supplementary material; Supplementary Table S1) were 5′-labeled with^32^ P-ATP using T4-polynucleotide kinase (New England Biolabs). Membranes were pre-hybridized for 10 min at 42°C before probing with 5′-labeled oligos overnight. Probed membranes were washed once in 2× SCC and 0.1% SDS for 10 min followed by a 10 min wash in 0.5× SCC and 0.1% SDS. Probed membranes were visualized by phosphorimaging on a Typhoon scanner (GE Healthcare).

### Complement deposition assay

D39V WT was inoculated to a start OD_600_ of 0.05 in CDM, CDM + 20% v/v plasma, CDM + 20% v/v heat-inactivated plasma or 100% CDM. After 1 h incubation at 37°C with 5% CO_2_, samples were harvested by centrifugation. Cell pellets were washed twice in 1xPBS and resuspended in SDS sample buffer [3% SDS, 10% glycerol, 50 mM Tris–HCl pH 6.8, 0.1% bromophenol blue, 12.5 mM EDTA, 5 mM dithiothreitol (DTT)] to a concentration of approximately 5*10^5^ cells/μL and heated at 90°C for 5 min. Samples were separated on a NuPAGE 4–12%, Bis-Tris gel (Invitrogen) for 45 min at 200 V in 1xMOPS running buffer, with a PageRuler Plus Prestained Protein Ladder, 10 to 250 kDa (Thermo Scientific). Separated proteins were transferred to a PVDF membrane in a wet tank transfer unit for 1 h at 300 mA. Blocking was carried out in 1xPBS-Tween (0.1% TWEEN20) with 5% w/v nonfat dry milk. Polyclonal rabbit anti-human C3c primary antibodies (Agilent / Dako) were used in a 1:10000 dilution (in 1xPBS-T with 2% w/v nonfat dry milk), while horseradish peroxidase (HRP)-conjugated polyclonal goat anti-rabbit secondary antibodies (Agilent/Dako) were used in a 1:4000 dilution. Washes with 1xPBS-T was carried out in between steps. Immobilon^®^ Forte was used as HRP substrate, and detection of chemiluminescent signals was carried out on an Amersham ImageQuant 800. As a loading control, GroEL was detected using polyclonal rabbit α-GroEL antibodies (Sigma-Aldrich, diluted 1:50000).

## Results

### Growth and survivability of *Streptococcus pneumoniae* in human blood samples and CSF

**Figure 1:**
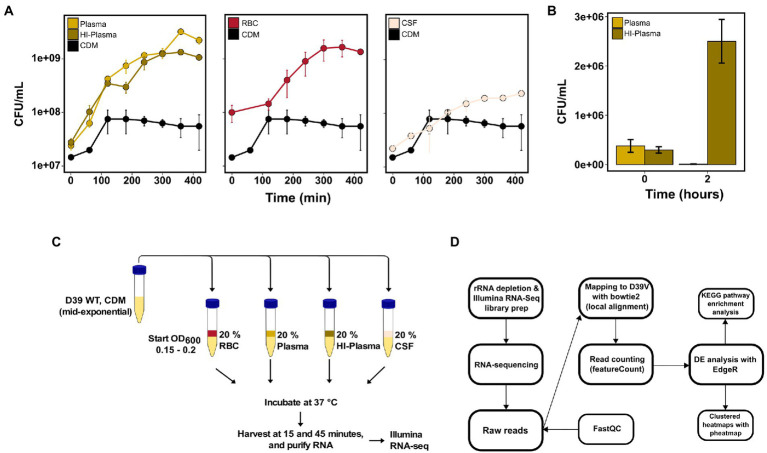
Pneumococcal *in-vitro* growth in invasive infection-associated human body fluids and RNA-sequencing workflow. **(A)** Mean CFU counts from 2 independent biological replicates of *S. pneumoniae* D39V grown in CDM compared to CDM supplemented with 20% pooled human plasma, heat-inactivated plasma (HI-plasma), red blood cells (RBC) or cerebrospinal fluid (CSF). Error bars represent standard deviations. **(B)** Heat-inactivation of plasma was validated by a plasma survival assay of the serum-sensitive *E. coli* NU14. Each bar represents mean CFU of *E. coli* after 0 or 2 h of incubation in 20% plasma and HI-plasma from 3 independent biological replicates. Error bars represent standard deviation. **(C)** For RNA-sequencing, *S. pneumoniae* D39V was grown in 20% plasma, HI-plasma, RBC, and CSF for 15 and 45 min before harvest. **(D)** Paired-end Illumina RNA-sequencing and analysis was carried out as described in the workflow.

Before determining the transcriptional adaptive responses of pneumococcus to components of the human blood and CSF, we first set out to examine the growth and survivability *in-vitro*. The wild-type *S. pneumoniae* D39V strain was grown in CDM with 0.5% glucose to the exponential growth phase, followed by transfer to 20% dilutions of plasma, heat-inactivated plasma (HI-plasma), RBCs and CSF in CDM, to a start OD_600_ of 0.02. The human blood and CSF samples were pooled samples collected from 28 donors (see *Materials and methods* for description of human blood and CSF collection). Samples for colony-forming unit (CFU)-counting were taken every hour for 7 h. All cultures were able to grow in the presence of the human blood components and CSF and reached a higher cell count compared to the control CDM culture (Figure 1A)—most likely due to additional nutrients supplied by the respective human samples. It is evident that pneumococci grow similarly in both plasma and HI-plasma, which was expected as pneumococcus and other gram-positive bacteria are resistant to the complement membrane attack complex (MAC). The human plasma was indeed active as shown in the killing assay with the serum-sensitive *Escherichia coli* strain NU14 (Figure 1B).

### Transcriptional response of pneumococcus to human blood components and CSF

To pinpoint the transcriptional responses of pneumococcus to human blood and CSF, D39V WT was incubated for 15 or 45 min in 20% dilutions of plasma, HI-plasma, RBCs, and CSF (Figure 1C). The early timeframe was chosen to observe the immediate responses caused by the human samples, and limit chances of effects caused by differences in growth states. Conversely, the 45-min incubation point was chosen to observe the adaptive response to the different blood components and CSF. Using blood fractions, rather than whole blood, allowed us to distinguish the response activated by non-cellular components present in plasma and the RBCs. Furthermore, plasma heat-inactivation allowed us to distinguish the response activated specifically by the active plasma proteins (e.g., complement proteins) from the rest of the plasma components. Despite being MAC-resistant, pneumococcus is still opsonized for phagocytosis *via* C3 deposition on the surface of the bacterium (Supplementary Figure S1; Hyams et al., 2010b; Pathak et al., 2018). We hypothesized that this complement-mediated opsonization might still trigger an adaptive transcriptional response in pneumococcus.

**Figure 2:**
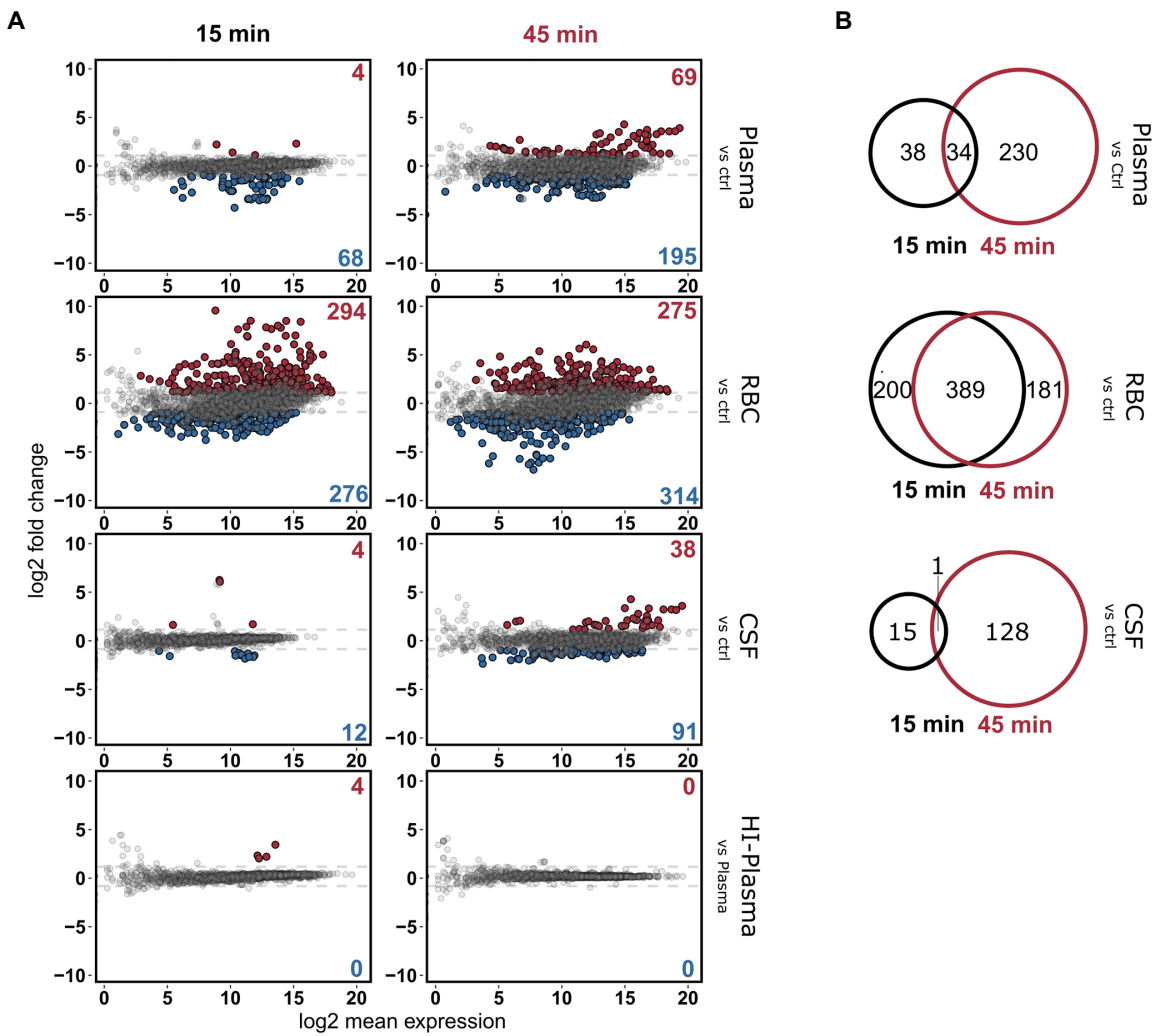
**(A)** MA-plots depicting the differentially expressed pneumococcal genes in 20% plasma, RBC and CSF cultures compared to the control CDM culture, at the two time points, 15 and 45 min. Differentially expressed genes in HI-plasma were also compared plasma. Red and blue dots represent significantly up-and downregulated genes, respectively [log_2_(FC) > 1 and FDR < 0.01]. **(B)** Veen-diagrams show the overlap in differentially expressed genes between the two time points.

RNA from these conditions was sequenced using Illumina RNA-sequencing and analyzed as depicted in the workflow (Figure 1D). Rapid transcriptional responses were observed for all conditions but was most pronounced for the bacteria incubated with RBCs where approximately a quarter of all pneumococcal genes were found to be differentially expressed (Figure 2A). Interestingly, a relatively large proportion of genes that were significantly differentially expressed at the earliest time point (15 min) were not differentially expressed at the later time point (45 min), and vice versa (Figure 2B). This shows that rapid, and transient, transcriptional responses occur that might be important for the ability of the pneumococcus to adapt to these conditions. The majority of overlapping genes, observed to be differentially expressed at both time points, was regulated in the same direction. The only exemption was *vanZ* in the plasma samples (downregulated at 15 min, and upregulated at 45 min), and *qsrB*, *purC* and *ribF* in the RBC samples (upregulated at 15 min, and downregulated at 45 min). Also, when comparing the transcriptional response of pneumococcus incubated in HI-plasma versus normal plasma, no clear differences in the early transcriptional response were observed. Only four genes were identified as significantly differentially expressed at 15 min, and zero at 45 min. These four gene are all part of the divergent *scr* operon involved with sucrose metabolism. The biological significance of this difference remains unclear. Nonetheless, it is evident that active immune components of the plasma do not induce any conspicuous transcriptional response in pneumococcus—at least not in the early stages of interaction. This contradicts the hypothesis stated earlier, that complement deposition would trigger a transcriptional response in pneumococcus, however that is not the case under these conditions.

**Figure 3:**
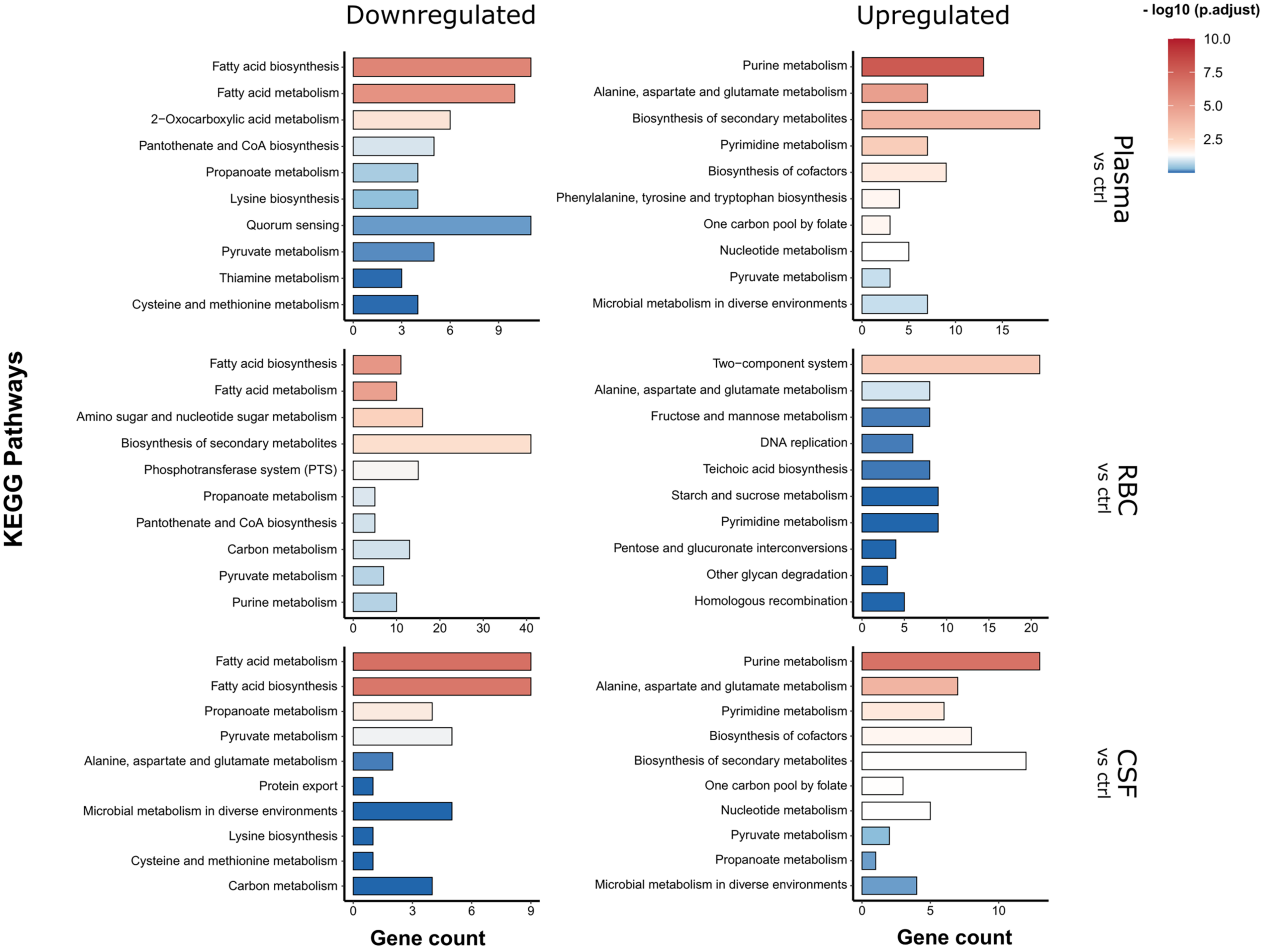
KEGG pathway enrichment analysis. Analysis was carried out on significantly differentially expressed genes [log_2_(FC) > 1 fold and FDR < 0.01]. KEGG pathways significantly enriched in the pool of up-or downregulated genes compared to the full genome background are represented by a red color hue, and non-significantly enriched pathways are represented by a blue color hue.

**Figure 4:**
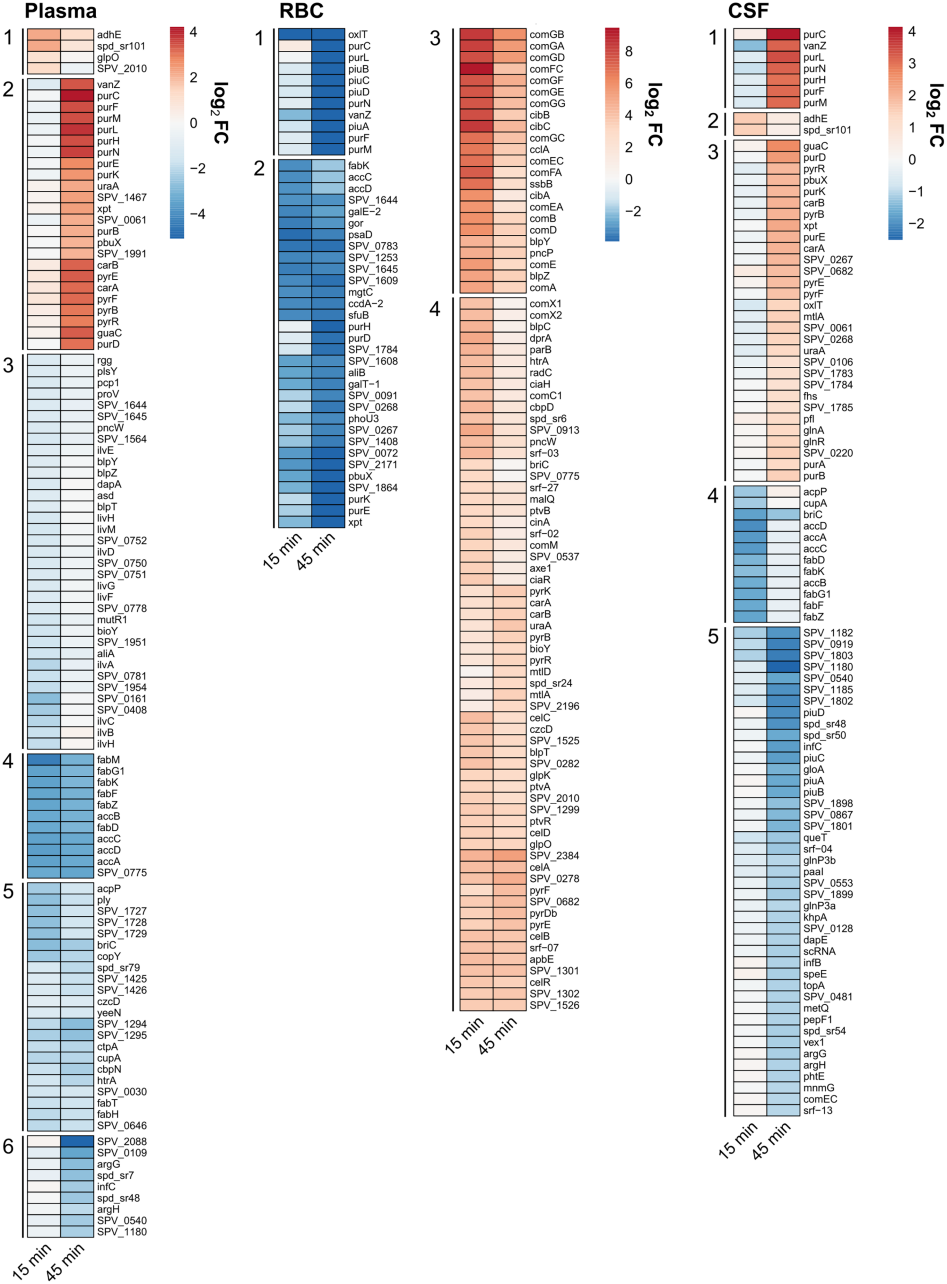
Clustered relative expression heatmaps showing the top differentially expressed genes in the different conditions: plasma, RBC and CSF cultures [FDR < 0.01, log_2_(FC) > 2 or log_2_(FC) < −2 for plasma, log_2_(FC) > 3 or log_2_(FC) < −3 for RBC, and log_2_(FC) > 1 or log_2_(FC) < −1 for CSF]. Clusters with a similar expression pattern from each condition were enumerated.

To determine which genetic and metabolic pathways that are differentially expressed in the different conditions, a KEGG Enrichment analysis was carried out using the clusterProfiler package (Figure 3). In short, the enrichment analysis identifies which KEGG pathways are overrepresented among the list of differentially regulated genes. All genes that were either significantly (FDR < 0.01) down- or upregulated [log_2_(FC) > 1] in either the 15- or 45-min samples were included in these analyses. This was to get a more comprehensive overview of all regulated pathways in a time-independent manner. In addition, an expression heatmap showing top significantly differentially expressed genes (clustered according to expression pattern) was created (Figure 4; Supplementary Figure S2A) to highlight the temporal differences in expression. A full list of differentially expressed genes can be found in Supplementary Table S2.

### Pneumococcal genes differentially regulated by human plasma

For the human plasma condition, the most apparent observation was that several genes involved with fatty acid biosynthesis/metabolism pathways were downregulated, whereas several genes involved with purine and pyrimidine metabolism pathways were being upregulated (Figure 3). From the heatmap it is evident that several genes that are part of the type II fatty acid synthesis (FASII) pathway (e.g., *fabK*, *fabD*, *fabG1*, *fabF*, etc.) were strongly downregulated (log_2_(FC) > 3) already after 15 min and continued to be downregulated after 45 min (Figure 4, plasma - cluster 4). Downregulation of genes involved in fatty acid biosynthesis is not surprising, since pneumococcus is capable to shut down *de novo* biosynthesis of fatty acids in favor of exclusively using exogenous fatty acids for phospholipid synthesis (Yao and Rock, 2017; Gullett et al., 2019), which are present in high levels in human plasma compared to other niches (Jumpertz et al., 2012; Abdelmagid et al., 2015). Previous studies have shown that the gene encoding the competence-induced peptide BriC, which promotes biofilm formation and nasopharyngeal colonization, is also co-transcribed with the *fab* gene cluster (Aggarwal et al., 2021). Our data support these findings, as we see a concurrent expression profile between *briC* and the *fab* genes (Figure 4; Supplementary Figure S2). Similarly, the gene encoding for the major pneumococcal virulence factor pneumolysin (*ply*) also showed a concurrent expression pattern with *acpP* and the rest of the *fab* genes (Figure 4, plasma - cluster 5; Supplementary Figure S2B). This is intriguing, as it suggests that a link between transcriptional regulation of fatty acid biosynthesis genes and *ply* might also exist.

The KEGG Enrichment analysis suggested that genes involved with purine and pyrimidine metabolism were upregulated during incubation with plasma (Figure 3). Looking at the expression heatmap we see a strong induction of the genes involved with *de novo* biosynthesis of purines and pyrimidines after 45 min of incubation (Figure 4, plasma - cluster 2). This could be a result of an insufficient supply of nucleotides from the media as the demand increases due to faster replication of cells in the plasma culture compared to the control CDM culture, as demonstrated in the growth assay (Figure 1A). However, this is not entirely consistent with the CSF culture, where pneumococcus did not replicate noticeably faster than in the CDM culture, and where upregulation of *de novo* nucleotide biosynthesis genes was also observed (Figure 4, CSF - cluster 1 and 3). Regardless of the cause for this upregulation, the data were consistent with what has previously been established in pneumococcus and other pathogenic bacteria, that *de novo* nucleotide biosynthesis is essential to pathogenicity and bacterial growth in nucleotide-limiting niches, such as the bloodstream and CSF (Hava and Camilli, 2002; Samant et al., 2008; Connolly et al., 2017; Goncheva et al., 2022).

Other notable genes being differentially regulated in plasma include virulence genes *cbpN (pcpA)*, *htrA*, and genes encoding transition metal transporters (*czcD*, *ctpA* and *cupA*; all downregulated at both 15 and 45 min). Several genes involved with peptide and amino acid metabolism and uptake were also weakly downregulated at 15 min (*ilvBHCA*, *livHMGF*. *proV*, *pcp1*, *aliA* etc.; Figure 4, plasma – cluster 3).

### Pneumococcal genes differentially regulated by human RBCs

**Figure 5:**
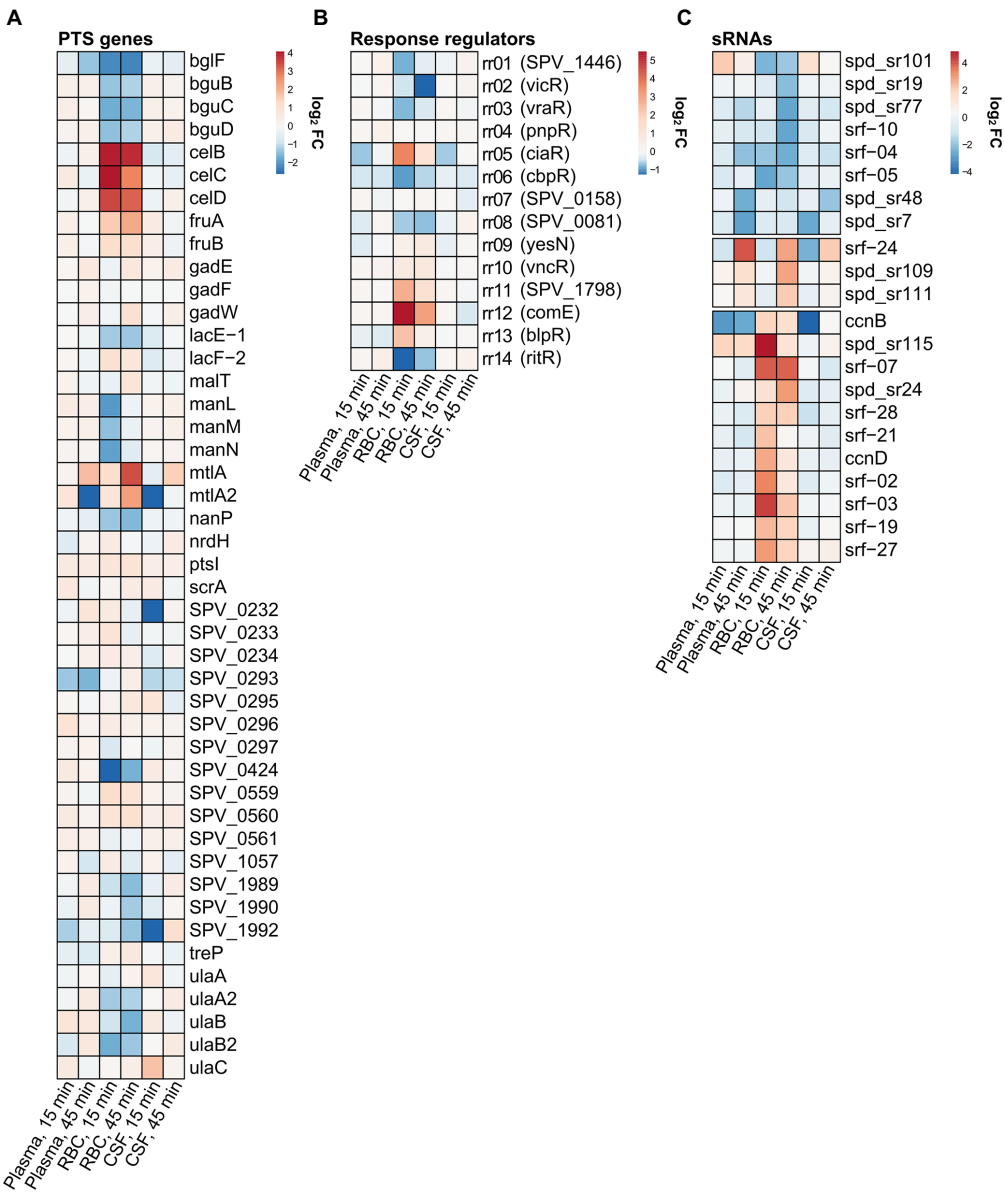
Relative expression heatmaps of **(A)** phosphotransferase systems (PTS) genes, **(B)** response regulators and **(C)** small non-coding RNAs. The lists include all PTS genes and response regulators annotated by KEGG. For sRNAs, only significantly differentially expressed sRNAs were included.

For the human RBC condition, the fatty acid biosynthesis pathway was also among the most downregulated pathways (Figure 3), which was expected since some of the fatty acids present in the human plasma most likely end up in the RBC fraction as well. Other notable KEGG pathways being downregulated include ‘biosynthesis of secondary metabolites’, ‘amino sugar and nucleotide sugar metabolism’ and ‘phosphotransferase systems (PTS)’. All these KEGG pathways include genes involved with a broad network of metabolic pathways and sugar utilization, and their enrichment suggests that an extensive rewiring of pneumococcal metabolism takes place during contact with RBCs. In fact, 23 of 45 pneumococcal PTSs were differentially expressed during contact with RBCs (Figure 5A). This included PTSs involved with utilization of a diverse set of sugars (e.g., cellobiose (*celBCD*, *bguBCD*), mannitol (*mtlA*, *mtlA2*), beta-glucosides (*bglF*), fructose (*fruAB*), lactose (*lacE-1*, *lacF-2*) and L-ascorbate(*ulaABC*)). The only significantly enriched pathway among the upregulated pathways was the ‘Two component-system’ pathway, which includes histidine kinases, response regulators and associated genes. Based on the expression heatmaps it is apparent that several response regulators were differentially expressed (Figures 4, 5B - RBC) in the RBC conditions. This is in stark contrast to the plasma and CSF samples, where no response regulators were significantly induced or repressed, further emphasizing that transcriptional rewiring is much more pronounced in the RBC condition. Many of these TCSs are related to pneumococcal competence, including *comE*, *blpR*, and *ciaR* (Slager et al., 2018b). In general, competence seems to be highly induced during incubation with RBCs, based on the numerous upregulated competence genes (Figure 4 - RBC, cluster 3). This is very much an indicator that RBCs induces a stress response in pneumococcus, as competence is not only involved with transformation, but is also a general stress response of pneumococcus (Claverys et al., 2006).

Another set of strongly downregulated genes was the RitR*-*regulated operon *piuBCDA* (Figure 4, RBC - cluster 1), which is involved in iron uptake and homeostasis (Ulijasz et al., 2004; Zhang et al., 2020). Even though iron is required for growth in the bloodstream, too much iron can also be toxic for cells, as ferrous iron (Fe^2+^) can interact with H_2_O_2_ (produced endogenously by pneumococcus) and result in formation of lethal hydroxyl radicals (Andrews et al., 2003). Decreased expression of the *piu* operon is therefore most likely a result of iron overload caused by heme-release from lysed RBCs.

Nucleotide biosynthesis genes were also significantly differentially expressed in the RBC condition. However, contrary to the plasma condition, purine biosynthesis genes were strongly downregulated after 45 min of incubation (Figure 4, RBC - cluster 1 and 2). This could indicate that RBCs contain a source for purines, thereby allowing for pneumococcus to downregulate *de novo* biosynthesis. In fact, RBCs are known to store large amounts of ATP that gets released to the bloodstream during hemolysis (Ferguson et al., 2021). The observed downregulation of purine biosynthesis genes might be a result of pneumococcus being able to take up and use the RBC-derived ATPs as a purine source.

### Pneumococcal genes differentially regulated by human CSF

For the human CSF condition, the most significantly enriched downregulated KEGG pathways were the fatty acid metabolism and biosynthesis pathways, while the most significantly upregulated KEGG pathways were the purine and pyrimidine metabolism pathways (Figure 3), as commented on in a previous section.

The downregulation of genes involved with fatty acid biosynthesis may be a direct result of the presence of fatty acids in the CSF samples. Compared to blood plasma levels, the normal levels of fatty acids in CSF are known to be very low but may increase in patients experiencing hemorrhagic strokes (Pilitsis et al., 2003). However, the fact that significant downregulation of *fab* genes is only observed after 15 min (Figure 4 – CSF, cluster 4), and to a low degree, is consistent with the suspected low levels of fatty acids in CSF.

**Figure 6:**
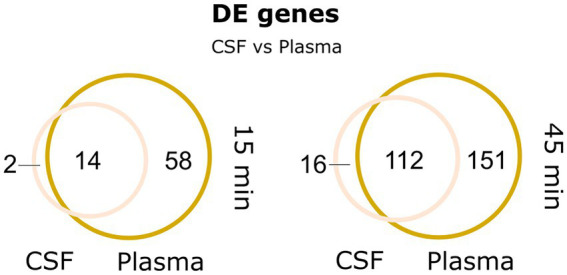
Venn-diagrams depicting the large overlap in differentially expressed genes between plasma and CSF at 15 and 45 min.

Interestingly, the transcriptional response in pneumococcus to CSF was very similar to the response to plasma, as illustrated in the Venn diagram (Figure 6) and by the expression heatmap of all differentially regulated genes (Supplementary Figure S2). In fact, at the 15 min time point, 14 out of 16 differentially expressed genes were also found differentially expressed in plasma, and at 45 min, it was 112 out of 129 differentially expressed genes. This is not surprising as CSF is frequently described as an ultrafiltrate of plasma and contains many of the same components. Interestingly, the most conspicuous CSF-specific transcriptional response, which was not observed for plasma, was the strong downregulation of *piuBCDA* operon at 45 min. As previously described, the *piuBCDA* operon is regulated by the RitR orphan response regulator in response to iron-and H_2_O_2_-mediated oxidative stress (Ulijasz et al., 2004). CSF is known to contain very low levels of iron, and it is therefore unlikely that iron-mediated oxidative stress is the cause for this downregulation. We did not observe the same concurrent downregulation of *ritR* in CSF, as we observed in the RBC condition (Figure 5B). This could indicate that another transcriptional regulator is involved.

### Several small non-coding RNAs are differentially expressed in human blood samples and CSF

**Figure 7:**
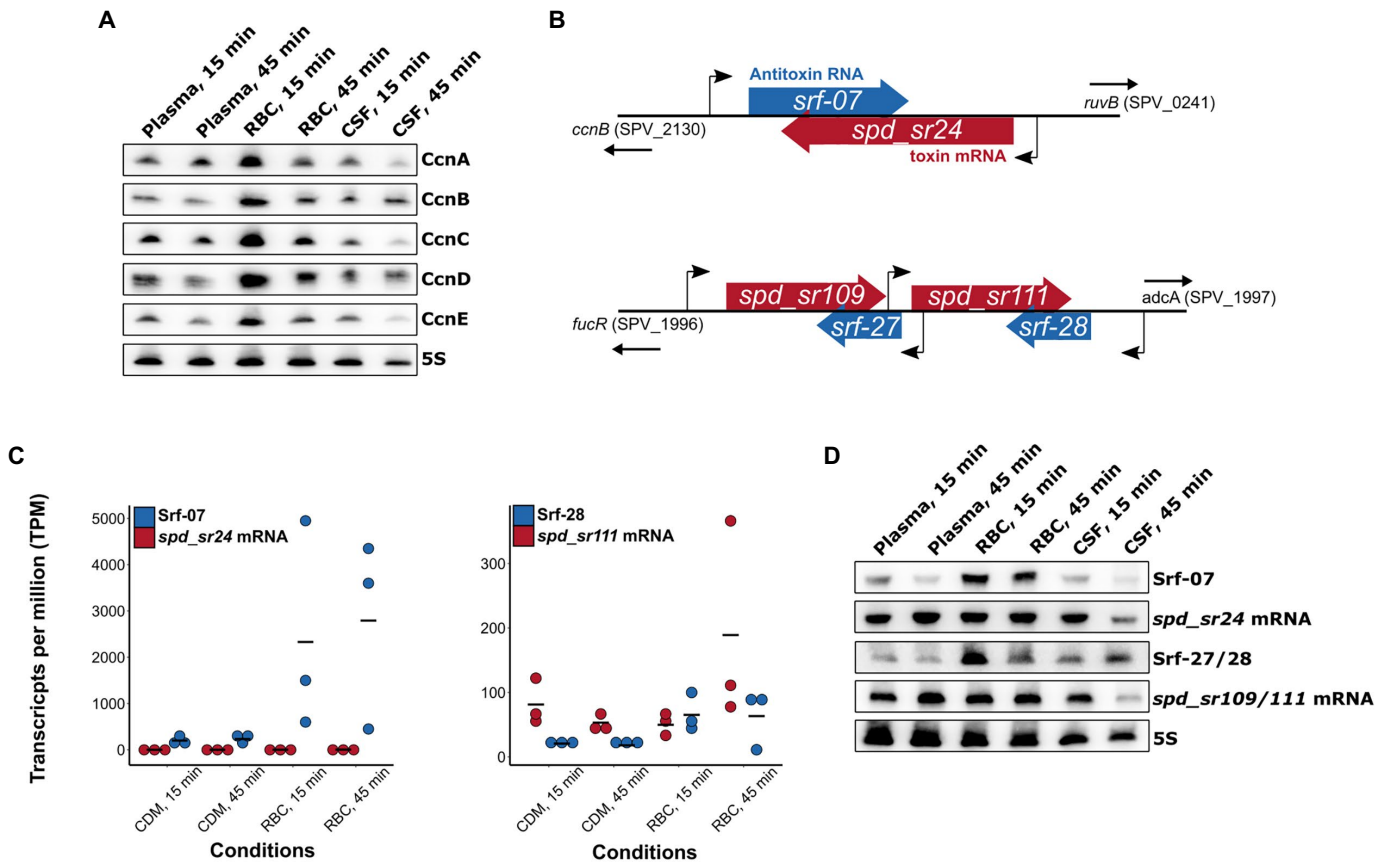
Validation of differentially expressed sRNAs. **(A)** Despite only CcnB and CcnD being identified as significantly differentially expressed in RBC cultures, all CiaR-dependent small RNAs (CcnA-E) showed a similar expression pattern, as expected. **(B)** Genetic context of differentially expressed RNAs belonging to the two pneumococcal Fst-like type I toxin-antitoxin (TA) loci. **(C)** Transcripts per million (TPM) calculations based on the RNA-seq data show a general molar excess of antitoxin RNA in the Srf-07/spd_sr24 TA system, but not in the Srf-28/spd_sr111 nor Srf-27/spd_sr109 TA systems. **(D)** Validation by northern blot (NB) analysis of toxin-and antitoxin RNA expression patterns from RNA-seq experiments. Expression pattern observed by NB analysis of antitoxin mRNAs were not consistent with RNA-seq data.

A field of gene regulation, which is incompletely described in pneumococcus is the ncRNA-mediated control of gene expression. In this analysis we wanted to get an overview of possible small ncRNAs (sRNAs) involved with gene regulation in relation to pneumococcal adaptation to human blood and CSF. Studies have already identified numerous potential regulatory sRNAs in *S. pneumoniae* D39 (Tsui et al., 2010;Slager et al., 2018a; Sinha et al., 2019). In this study we focused on the 30 sRNAs already annotated in D39V (Slager et al., 2018a), and manually added 74 sRNAs annotated by Sinha et al. in D39W (Sinha et al., 2019). A total of 22 sRNAs were found to be differentially regulated [log_2_(FC) > 2 or log_2_(FC) < −2] across the different conditions, with the majority originating from the RBC condition (Figure 5C). Two of these are previously described members of a group of 5 sRNAs regulated by the response regulator CiaR (CcnB-and D; Marx et al., 2010). Like CiaR, these sRNAs were highly induced at the earliest time point (15 min) in RBC, and less induced at the later time point (45 min; Figures 5B,C). Surprisingly, only 2 out of 5 CiaR-regulated sRNAs were identified as differentially expressed based on the RNA-seq analysis, however, northern blot analysis reveals that all the CiaR-regulated sRNAs follow a similar expression pattern (Figure 7A). A recent new member of the CiaR regulon, Srf-21 (Slager et al., 2018b), was also revealed by the RNA-seq data to be differentially regulated in RBC, following the same pattern as the other CiaR-regulated sRNAs, further validating it as a CiaR-regulated sRNA.

None of the remaining 20 differentially regulated sRNAs have previously been described in literature. Based on information available from PneumoBrowse and the study from Sinha et al. (Slager et al., 2018a; Sinha et al., 2019), the 22 sRNAs were divided into different groups (Table 1). 15 of the 22 sRNAs were found to be located inside mRNAs, primarily in untranslated regions (either the 5’-UTR, 3’-UTR or overlapping both). 5′- and 3’UTR-located ncRNAs are very often involved with *cis*-acting regulation (e.g., thermoswitches, and small-molecule riboswitches), however, in recent years 3’UTRs have been recognized as a large reservoir for *trans*-acting sRNAs (Menendez-Gil and Toledo-Arana, 2021). Two sRNAs were predicted to be the RNA leader sequences of the ribosomal protein-encoding gene *rpsF* and the translation initiation factor-encoding gene *infC*. Only four of the sRNAs were predicted to be transcribed from intergenic regions, and 3 of these were the previously described CiaR-regulated sRNAs. These data suggest a limited involvement of the classical group of intergenic, *trans*-acting sRNAs, and a larger potential involvement of UTR-derived sRNAs in pneumococcal gene regulation.

**Table 1:** Differentially expressed small ncRNAs identified in this study divided into groups based on information from literature or genetic context.

sRNA Group^1^	Differentially expressed sRNAs
CiaR-regulated sRNAs	CcnB, CcnD, Srf-21
Intergenic	Srf-05
5’-UTR-located sRNAs^2^	Srf-03 (*comA*), Srf-19 (*SPV_1137*), Srf-10 (*bglG*, antiterminator), Spd_sr19 (*SPV_0131*)
3’-UTR-located sRNAs^2^	Spd_sr101 (*adhE*), Spd_sr7 (*SPV_0803*), Srf-02 (*SPV_0047*), Spd_sr115 (*SPV_2455*)
5′/3’-UTR-located sRNAs^2^^,^^3^	Srf-24 (*hexA/patA*), Srf-04 (*mnmA/ mnmG*)
TA system RNAs (type I)	Srf-07, Srf-27, Srf-28 (RNA antitoxins), Spd_sr24, Spd_sr109, Spd_sr111 (toxin-encoding mRNAs)
RNA-switches^2^	Spd_sr48 (*infC*, RNA leader), Spd_sr77 (*rpsF*, RNA leader)

1 Based on previous studies, or predicted from available data [PneumoBrowse, or Sinha, Dhriti et al. (Sinha et al., 2019)].

2 Associated genes in parenthesis.

3 sRNAs with a shared predicted promoter with the downstream gene and shared predicted transcriptional terminator with upstream gene.

Six sRNAs belonging to 3 sets of predicted type I Fst-like toxin-antitoxin (TA)-systems were all identified as differentially expressed (Table 1; Figure 5C). TA-systems are regulatory systems involved with a wide range of bacterial functions such as plasmid stability, stress-and starvation tolerance, and antimicrobial persistence (Jurėnas et al., 2022). Each of these uncharacterized type I TA-systems consist of an antisense RNA antitoxin (Srf-07, Srf-27 and Srf-28) and an associated toxin-encoding mRNA (*spd_sr24*, *spd_sr109* and *spd_sr111*). Note that the latter were identified as sRNAs in D39W in the study by Sinha et al. but are predicted to encode the following type I toxin ORFs annotated by PneumoBrowse in D39V: SPV_2132, SPV_2448, SPV_2450. The *srf-07/spd_sr24* TA locus is located upstream *ruvB*, while the *srf-27/spd_sr109* and *srf-28/spd_sr111* are *par* homologous TA-systems located in tandem downstream *fucR* (Figure 7B). According to the RNA-seq data, the RNA antitoxins were all induced during incubation with RBCs, while their respective toxin mRNA showed different expression patterns depending on the system (Figure 5C). The toxin-encoding *spd_sr24* mRNA was weakly induced by RBCs at both time-points, while the *spd_sr109* and *spd_sr111* mRNAs were only induced in RBC at the late time-point (45 min). Despite weak induction of the toxin *spd_sr24* mRNA was observed, the normalized transcript levels per million (TPM) showed consistent low levels of toxin mRNA compared to antitoxin RNA Srf-07 across all conditions (Figure 7C). RBC-specific induction of Srf-07 with consistent levels of toxin mRNA across the different conditions (except for CSF, at 45 min) was also confirmed by northern blotting (Figure 7D).

The *spd_sr109/111* mRNA levels were not consistent between the RNA-seq data and NB analysis (Figure 5C, 7D). However, for these *par* homologous TA systems, the toxin mRNA TPM were generally higher compared to antitoxin RNA TPM (Figure 7C). This was also supported by the NB analysis, where we noticed much stronger signals for the *spd_sr109/111* mRNA blot compared to Srf-27/28 blot. Unless other factors are involved, this might suggest that this system is ‘ON’ in all our tested conditions, as indicated by the molar excess of toxin mRNA.

Incubation with RBC evidently causes some potentially interesting regulatory events in the Fst-like TA systems of pneumococcus; however, it should be noted that the transcript levels are not necessarily a good indicator of TA activity (LeRoux et al., 2020), and further investigation into TA activity of these systems is definitely needed.

## Discussion

Invasive pneumococcal infections of the bloodstream and CSF often have fatal outcomes. For development of future treatment strategies, it is crucial that we understand pneumococcal invasive infection mechanisms and know how it adapts to the different niches of the human body. All studies so far investigating the pneumococcal transcriptional response in invasive niches such as the blood and CSF, have either used murine models or niche-mimicking conditions (Orihuela et al., 2004; Ogunniyi et al., 2012; Shenoy et al., 2017; Aprianto et al., 2018; D’Mello et al., 2020). For the first time, this study used RNA-sequencing to globally assess the transcriptional response of the pathogenic *S. pneumoniae* D39V strain to human blood components and CSF. This allowed us to get more detailed insight into human-specific interactions. By using pooled biological samples, the focal point of the study was the general transcriptional responses to human blood components and CSF and did not consider inter-individual differences in sample composition, and consequently potential differences in inter-individual susceptibility to invasive pneumococcal infection. Apart from a few exceptions, we did not observe any greater similarities in the expression patterns observed in our data with transcriptomic data obtained from similar conditions in previous mice studies (Orihuela et al., 2004; Shenoy et al., 2017; D’Mello et al., 2020). This is most likely an effect of both the stark differences in models used, as well as host species-specific responses.

In this study, we observed strong downregulation of genes involved with the FASII biosynthesis pathway in blood (both plasma and RBC conditions). Like other firmicutes, pneumococcus can acquire exogenous fatty acids for membrane phospholipid synthesis (Yao and Rock, 2017). Blood plasma contains relative high levels of fatty acids, and it is conceivable that pneumococcus, as a result, downregulate *de novo* fatty acid biosynthesis to instead utilize exogenous fatty acids for phospholipid synthesis. Interestingly, downregulation of fatty acid biosynthesis genes was also observed during infection of the bloodstream in mice, in studies by Shenoy et al. (2017), and D’Mello et al. (2020).

Our data also support previous findings that transcription of the competence-induced *briC* gene is co-transcribed with the upstream *fab* gene cluster (Aggarwal et al., 2021), as demonstrated by its concurrent expression pattern. BriC promotes biofilm formation and induces *fabT* transcription (Aggarwal et al., 2021). FabT is a transcriptional repressor of the *fab* genes and alters the balance of saturated / unsaturated fatty acids in the membrane, as well as influences colony phase variation (Jerga and Rock, 2009; Aggarwal et al., 2021; Zhang et al., 2021), which is an important regulatory element of pneumococcal virulence. Our RNA-seq data also showed a concurrent expression pattern between the gene encoding the major virulence factor Ply, *acpP* and most of the *fab* genes. Transcriptional regulation of *ply* has not previously been described; however, this data suggests that it is coupled to the regulation of fatty acid biosynthesis. Downregulation of *ply* in human blood is also consistent with above-mentioned studies that showed downregulation of *fab* genes *in-vivo* in the bloodstream of mice (Shenoy et al., 2017; D’Mello et al., 2020). In summary, the RNA-seq data imply that fatty acids within the bloodstream serve as extrinsic signaling molecules important not only for regulating pneumococcal fatty acid biosynthesis and membrane lipid composition but also for key virulence attributes such as colony phase variation and virulence gene expression.

We did not observe clear differences when comparing the pneumococcal response to plasma and heat-inactivated plasma, indicating that the active immune components of the blood (i.e., the complement proteins) have no immediate impact on the transcriptional response in pneumococcus. This finding is interesting, as it suggests that the pneumococcal defense against the complement system does not include a rewiring of transcriptional networks. Instead, our data suggest that pneumococcus is more responsive to metabolic components of the plasma (e.g., fatty acids and nucleotide base availability), and cellular components of the blood, such as the RBCs. As established earlier, a complex rewiring of the transcriptional network was observed after incubation with RBCs, with more than 500 genes being differentially expressed after 15 and 45 min, including changes to expression of *de novo* nucleotide biosynthesis genes, fatty acid biosynthesis genes, genes involved with transition metal transport (*piuBCDA*, *czcD*), phosphotransferase systems (e.g., cellobiose, mannitol, beta-glucoside and lactose-specific PTSs) and several competence genes [indicative of a general stress response (Claverys et al., 2006; Engelmoer and Rozen, 2011)]. Downregulation of the *piuABCD* operon was unexpected, as previous *in-vivo* transcriptomics data showed strong upregulation of *piuA* in the bloodstream of mice (Ogunniyi et al., 2012). We speculate that downregulation of this operon may reflect iron stress caused by extensive release of heme from lysed RBCs, which might not be as widespread *in-vivo* compared to *in-vitro*.

Lysed RBCs also release high levels of ATP (Sikora et al., 2014; Ferguson et al., 2021), which could serve as a purine source and cause the observed downregulation of purine biosynthesis genes after 45 min. Interestingly, the direct opposite was observed in plasma, which begs the question, would these responses cancel each other out in whole blood? Ultimately, it depends on multiple factors, including whether expression is strictly regulated by exogenous availability of purine sources. It is evident that differential regulation of purine-and pyrimidine biosynthesis genes might be more complex *in-vivo*. In fact, a previous microarray analysis of pneumococci isolated from the bloodstream of mice found purine biosynthesis genes to be upregulated, while pyrimidine biosynthesis genes were downregulated (Orihuela et al., 2004), which is directly opposite to what we observe for pneumococci incubated with human RBCs *in-vitro*. Upregulation of purine and pyrimidine biosynthesis genes has also been observed previously during contact with a human lung cell line (Aprianto et al., 2016), indicating that rewiring of these metabolic pathways might be a common stress response in various niches of the human host. In addition to serving as building blocks for DNA and RNA biosynthesis, nucleotide metabolism is linked to important aspects of virulence and stress adaptation. For example, pyrimidine metabolism has already been linked to capsule biosynthesis. Here pyrimidine nucleotides serve as precursors for activated nucleotide-sugars needed for synthesis of the capsule polysaccharides (Carvalho et al., 2018). Similarly, purine nucleotides serve as precursors for important second messengers involved with stress responses like ppGpp and cyclic di-AMP (Kazmierczak et al., 2009; Corrigan and Gründling, 2013; Zarrella et al., 2020). It would be interesting to further uncover how the changes to expression of nucleotide biosynthesis genes are intertwined with the pneumococcal stress responses and virulence during host niche adaptation.

To get a full understanding of pneumococcal adaptation it is important to identify the differentially regulated pathways as well as the regulatory cellular components that regulate these changes. In this study, we focused on identifying potential regulatory sRNAs, and found 22 pneumococcal sRNAs to be differentially expressed across the different conditions. This included the CiaR-regulated sRNAs, *CcnA-E*, RNAs belonging to type I TA-systems, and numerous uncharacterized sRNAs. Our knowledge about the biological functions of the Fst-like TA-system RNAs in pneumococcus and other bacterial species is still limited (Weaver, 2020). Some Fst-like toxins disrupt cell membrane integrity, while others do not. Curiously, two toxins from the Fst/Ldr-like superfamily in *Staphylococcus aureus*, PepA1 and PepA2, are both known to be effective at lysing RBCs, indicating that they might have a dual mode of action against both bacteria and host cells during invasive infections (Sayed et al., 2011; Germain-Amiot et al., 2019). A similar dual function could potentially be performed by the Fst-like toxin of pneumococcus. How this would correlate with the observed transcriptional changes to the TA-system RNAs remains to be determined. Although transcript levels are not necessarily a precise indicator of TA-activity, it was quite interesting to observe that the *par* homologues type I TA-systems (*srf-27/spd_sr109* and *srf-28/spd_sr111*) showed substantially higher levels of the toxin mRNA compared to its antitoxin RNA, even in non-stressed conditions. Unless other factors are involved, this could indicate that toxin expression occurs even in non-stressed conditions, which is atypical for TA-systems (Jurėnas et al., 2022).

Our bioinformatic analysis demonstrated that most of the uncharacterized sRNAs are located within UTRs of mRNAs, suggesting that they could be processed regulatory sRNAs or *cis*-acting elements, as previously demonstrated many times in the literature (Adams et al., 2021; Menendez-Gil and Toledo-Arana, 2021). A future study aimed at identifying the function of these RNAs, and their potential involvement in virulence, will not only improve our understanding of pneumococcal host-niche adaptation, but could potentially also advance our understandings of gram-positive ncRNA-mediated gene regulatory mechanisms, which are less well-described compared to those of gram-negative bacteria (e.g., Gammaproteobacteria).

While the transcriptomic analysis presented in this study reveal several interesting virulence-associated metabolic pathways and gene-regulatory networks to pursue in future studies, it would also be highly relevant for future studies to characterize the proteome of invasive pneumococci using a similar setup. In combination with a transcriptional analysis, this could reveal potential post-transcriptional regulatory mechanism important for host-niche adaptation, not discovered by a transcriptional analysis alone. Currently, a limited number of studies have investigated the proteome of pneumococci in response to invasive niches conditions (Bae et al., 2006; Bittaye et al., 2017).

In summary, these data revealed extensive changes to the pneumococcal transcriptome during incubation with human blood components and CSF. Extensive transcriptional changes were already observed at early time points, with the biggest changes observed during incubation with RBCs. This suggests that the cellular components of the blood are the main stressors to pneumococcus inside the bloodstream, and that the plasma components primarily render a metabolic adaptation response in pneumococcus. Interestingly, most of the changes observed for CSF samples, were also observed for the plasma samples, which is not surprising, as CSF shares many similarities with plasma. Additionally, several uncharacterized sRNAs were identified as differentially expressed in response to the different conditions – in particular to the RBCs. To fully understand the adaptive response of pneumococcus during invasive infections of the human bloodstream and CSF, these data suggest that future research should aim to further uncover the involvement of metabolic pathways such as fatty acid and nucleotide biosynthesis, as well as ncRNA-mediated regulation, in pneumococcal virulence.

## Data availability statement

The data presented in the study are deposited in the Gene Omnibus Expression (GEO) database repository, accession number GSE214359.

## Ethics statement

Cerebrospinal fluid (CSF) samples were obtained as leftover material from routine analysis of patient samples from Odense University Hospital provided by associate professor Louise Helskov Jørgensen. The samples were obtained fully anonymized, transferred to unlabeled tubes and pooled. The pooled samples were used for growth of bacteria. The use of leftover patient material for this purpose has been evaluated by the Regional Committees on Health Research Ethics for Southern Denmark (S-20212000 Acadre 21/35007).

## Author contributions

JP and MJ wrote the manuscript, while FH and MJ conceptualized the study. FH and JP contributed to the experimental work. JP and FN contributed to the bioinformatic analyses. MJ and JM-J supervised and funded the project. All authors edited or provided feedback for the manuscript and approved the submitted version.

## Funding

This project was funded by the Danish Research Council (FSS) through a Grant to JM-J and MJ (Grant no. 9040-00139B).

## Conflict of interest

The authors declare that the research was conducted in the absence of any commercial or financial relationships that could be construed as a potential conflict of interest.

## Publisher’s note

All claims expressed in this article are solely those of the authors and do not necessarily represent those of their affiliated organizations, or those of the publisher, the editors and the reviewers. Any product that may be evaluated in this article, or claim that may be made by its manufacturer, is not guaranteed or endorsed by the publisher.
