# Supplementary material for: Global transcriptional responses of pneumococcus to human blood components and cerebrospinal fluid
Source: Front Microbiol. 2022 Dec 21;13:1060583. doi: 10.3389/fmicb.2022.1060583 (PMC9812572; doi:10.3389/fmicb.2022.1060583)
Supplement: Supplementary file 1 [file Data_Sheet_1.PDF]

Supplementary Material

Table S1. Strains and oligos used in this study

**Strains**	
Streptococcus pneumoniae strain D39V	
Escherichia coli strain NU14	
**DNA oligos**	**Sequence (5’->3’)**
**ccnA_NB**	CACCTGATTGGGTGGCTTCATTAGGAGATTGTGATGAAAA
**ccnB_NB**	CCACCTGATTGGGTGGAGTTAAGGGAGATTATTATGAAAA
**ccnC_NB**	TTTAGGATATTTGTTACAACAAGTTAGGAGGTCTTCTTGTA
**ccnD_NB**	TGAAAAAGTTTTAGGAGTTTAAGTTAAGGTCTTCTTAACT
**ccnE_NB**	TAAATAAAGATATTAGATGAAAATCAAATTCAAACTAATTCAGT
**srf07_NB**	TTTGGTCGTGAGCTTGGGGTCTTTTCTAGCCTATGATAT
**Spd_sr24_NB**	TGTCCTTGTTTAGCCATTTATCGACTATACGAAGAACGAC
**Srf-27/Srf-28_NB**	CCTTAACTACGGCCATAGTTAGGGGCTTTGGTGTTCTAAT
**Spd_sr109/spd_sr111_NB**	AGTTAAGGGCTTTTTTGACGTATCTTAGCGATTTAACGGG
**5SrRNA_NB**	GTTTCACTTCTGAGTTCGGCATGGGGTCAGGTGGG

Table S2. Differential expression analysis of all genes with EdgeR (excel sheet uploaded separately).

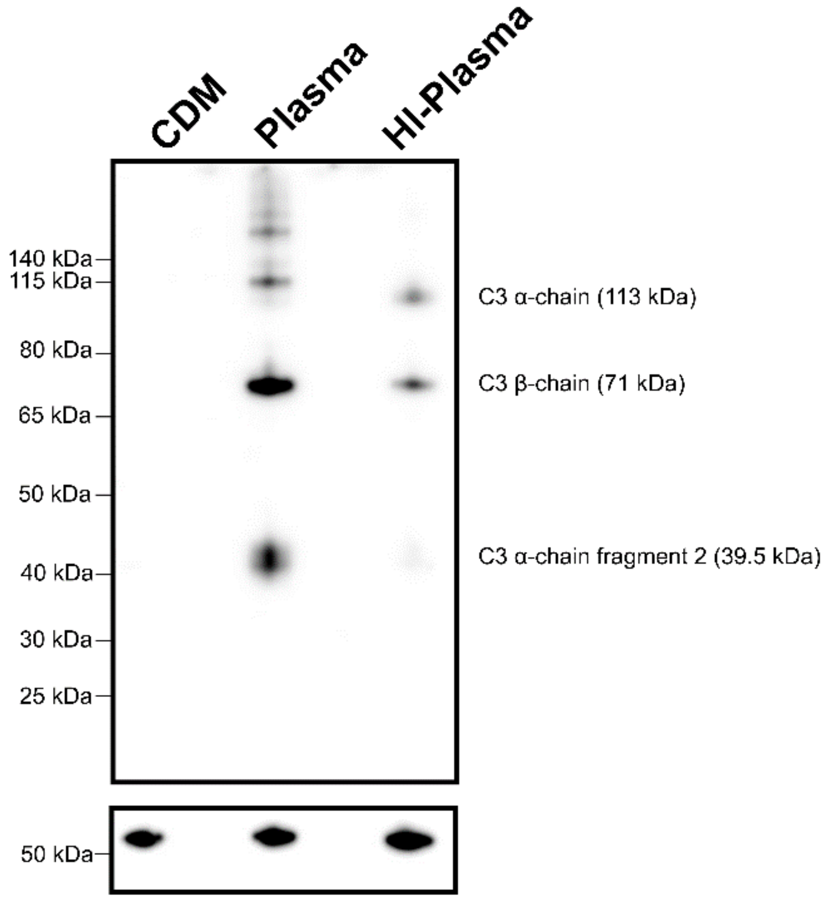

Supplementary Figure 1. Complement deposition assay with *S. pneumoniae* D39V incubated in CDM, 20 % plasma or 20 % heat-inactivated plasma (HI-plasma) for 1 hour at 37 °C. Bacterial lysates were separated by SDS-PAGE, and western blot analysis was carried out with anti-C3c antibodies. Substantially higher levels of C3 deposition were observed for pneumococcus incubated with plasma compared to HI-plasma. C3 fragments are estimated based on molecular weight.

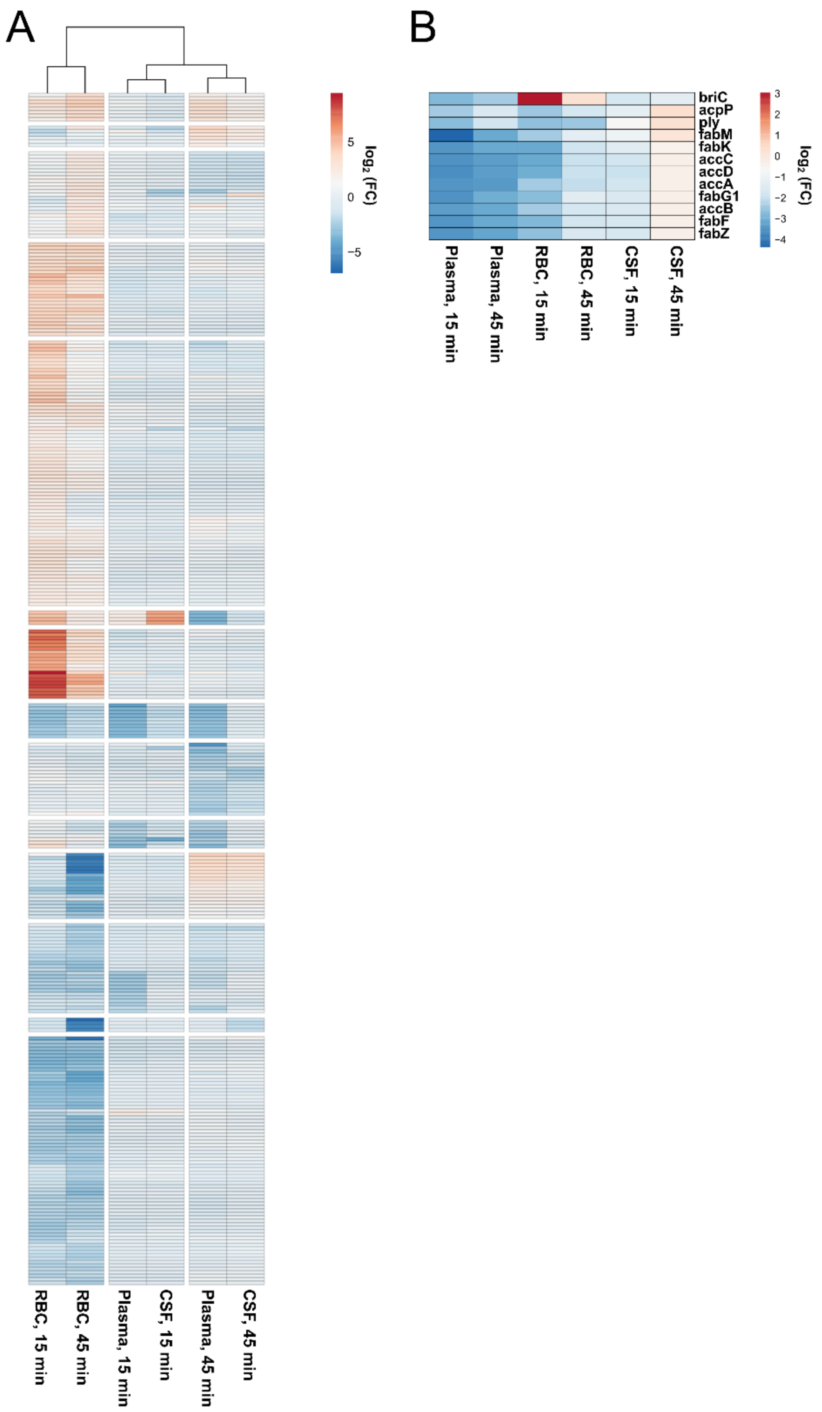

Supplementary Figure 2. (A) Clustered relative expression heatmaps showing all differentially expressed genes in plasma, RBC and CSF cultures (FDR<0.01, $\log_2$ (FC)>2 or $\log_2$ (FC)<-2). Both rows (genes) and columns (sample) are clustered, and it demonstrates that the expression pattern of most differentially expressed genes in CSF and plasma follow a similar expression pattern. (B) The relative expression heatmaps of selected fatty acid biosynthesis genes (*acpP*, *fabM*, *fabK*, *fabG1*, *fabF*, *fabZ*, and *accA-D*) compared to competence-induced peptide-encoding gene *briC* and virulence gene *ply*
